# Real-world experience of the efficacy and safety of guselkumab 100 mg in patients with palmoplantar pustulosis in Korea: A retrospective single-center study

**DOI:** 10.1016/j.jdin.2024.02.018

**Published:** 2024-04-07

**Authors:** Min Kyung Cho, Dong Hyun Kim

**Affiliations:** Department of Dermatology, CHA Bundang Medical Center, CHA University School of Medicine, Seongnam, Korea

**Keywords:** biologics, guselkumab, palmoplantar pustulosis

*To the Editor:* Treating palmoplantar pustulosis (PPP) is often challenging for dermatologists due to a lack of standard therapy and limited responses. However, guselkumab, an interleukin 23 blocker, has recently been utilized worldwide for PPP and was approved in Korea in 2019 for adult patients with moderate to severe PPP.^1^^,^^2^

We conducted an investigation into real-world data concerning the effectiveness and safety of guselkumab in patients with PPP who exhibited limited responses to conventional therapies. This study included total of 17 patients diagnosed with PPP who received more than 4 cycles of guselkumab 100 mg injection at CHA Bundang Medical Center between January 2018 and December 2023. The demographics and clinical characteristics are shown in Table I. Common comorbidities included hypertension (29.4%) and hyperlipidemia (23.5%), consistent with the previous reports.^1^^,^^3^ Additionally, latent tuberculosis was found in 35.3% of the patients during screening, necessitating concurrent antituberculosis treatment with biologics.

**Table I:** Characteristics of the included patients (*n* = 17)

Gender, *n* (%)	
Male	4 (23.5)
Female	13 (76.5)
Age, y, median (range)	46 (34-71)
Duration of PPP, y, median (range)	3 (2-12)
Ever-smoker, *n* (%)	11 (64.7)
Recurrent tonsillitis, *n* (%)	2 (11.8)
Initial PPPASI before guselkumab, mean (range)	13.3 (4.2-31.2)
Previous treatment, *n* (%)	
Systemic treatment	
Acitretin	16 (94.1)
Cyclosporine	15 (82.4)
Methotrexate	2 (11.8)
Biologics	0
Laser treatment	
NBUVB	2 (11.8)
Excimer laser	1 (5.9)
Concurrent treatment, *n* (%)	
Systemic treatment	
None	7 (41.2)
Acitretin	8 (47.1)
Cyclosporine	3 (17.6)

*NBUVB*, Narrow band ultraviolet light B; *PPP*, palmoplantar pustlosis; *PPPASI*, palmoplantar pustulosis psoriasis area and severity index.

For each patient, we measured disease severity using the palmoplantar pustulosis area and severity index (PPPASI). The median value of the initial PPPASI before starting guselkumab was 12.2, with a range from 4.2 to 31.2 (Fig 1) and the proportion of patients achieving PPPASI-50 and PPPASI-75 was 82.4% and 47.1%, respectively. Among them, 58.8% used concurrent systemic therapy with acitretin or cyclosporin.

**Fig 1:**
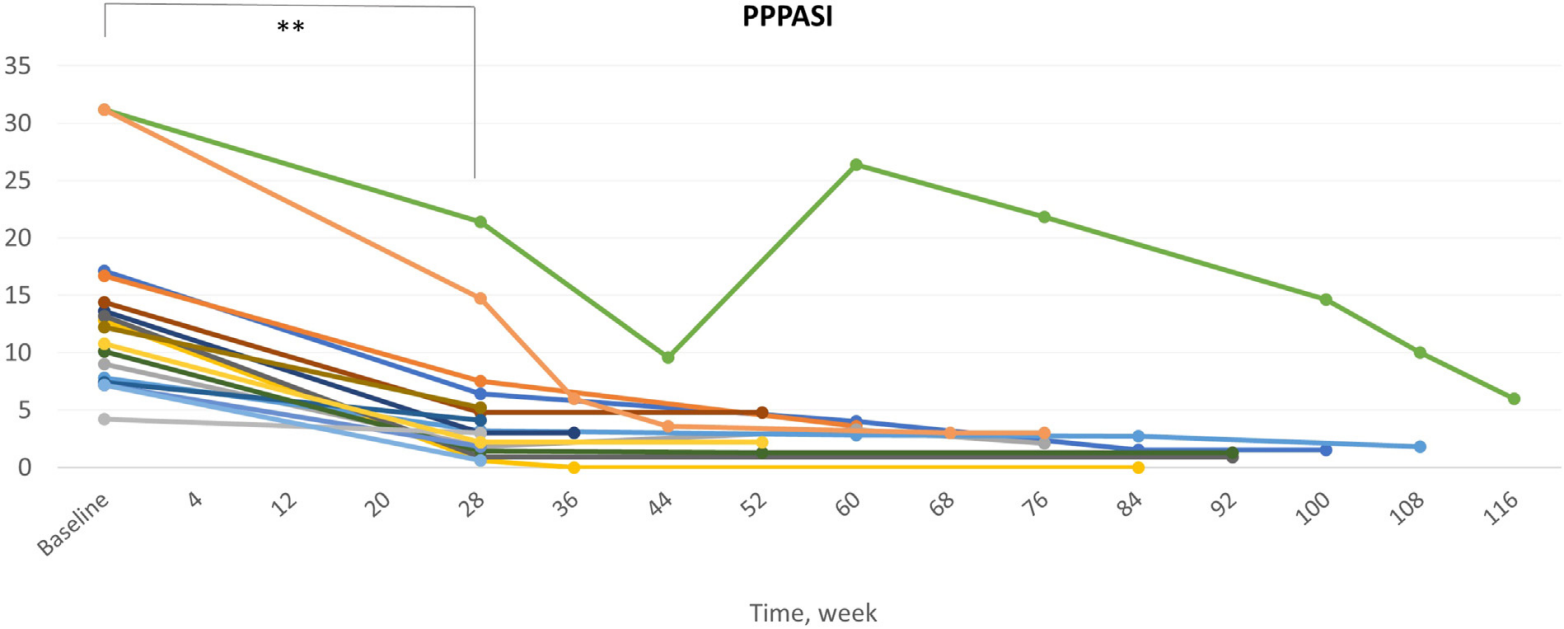
The entire course of PPPASI, with a maximum of 116 weeks. The median PPPASI score was significantly improved in 28 weeks. Wilcoxon’s signed rank test, ∗*P* < .05; ∗∗*P* < .01. *PPPASI*, Palmoplantar pustulosis psoriasis area and severity index. Each patient is marked with the different *color lines*.

Two cases of mild injection site reactions were reported, and neither of them discontinued the treatment due to the side effect. As of now, patients have been maintaining treatment for up to a maximum of 118 weeks without experiencing any further serious adverse events.

Due to its low prevalence, population-based studies on PPP are limited. In the previously reported Phase 3 study results of guselkumab from Japan, the PPPASI-50 achievement rate at week 24 was 79.6%, and the PPPASI-75 achievement rate was 27.8%,^4^ demonstrating further symptom improvement through week 60, with sustained efficacy observed until week 84.^1^ Erythema and pustules/vesicles responses were suggested as early indicators for assessing treatment efficacy.^4^

Additionally, we conducted a correlation analysis to identify factors influencing treatment response. However, only lower body mass index showed a significant positive correlation, while gender, age, baseline PPPASI score, duration of the disease, concomitant therapy, smoking and tonsilitis were all unrelated to treatment response. The result indicating an association between lower body mass index and better improvement is consistent with the previous report.^1^

Interesgingly, the treatment progress of 2 patients with the highest initial PPPASI of 31.2 showed notable differences (Fig 1). The patient who experienced a symptom flare-up during the course had recurrent tonsillitis and continued smoking. Although not statistically significant, the authors consider these factors may potentially impacted a slower response, as they have been mentioned as exacerbating factors in PPP.^5^

While this study has limitations due to the small number of patients from a single center, it holds significance as the first research conducted on Korean patients with PPP, rather than being a single case report. The favorable efficacy and safety profile of guselkumab offer a promising treatment alternative for patients with challenging PPP.

## Conflicts of interest

None disclosed.
